# Target Prediction Model for Natural Products Using Transfer Learning

**DOI:** 10.3390/ijms22094632

**Published:** 2021-04-28

**Authors:** Bo Qiang, Junyong Lai, Hongwei Jin, Liangren Zhang, Zhenming Liu

**Affiliations:** State Key Laboratory of Natural and Biomimetic Drugs, School of Pharmaceutical Sciences, Peking University, Beijing 100191, China; bqiang@bjmu.edu.cn (B.Q.); jylai@bjmu.edu.cn (J.L.); jinhw@bjmu.edu.cn (H.J.)

**Keywords:** target prediction, deep learning, transfer learning, natural product

## Abstract

A large proportion of lead compounds are derived from natural products. However, most natural products have not been fully tested for their targets. To help resolve this problem, a model using transfer learning was built to predict targets for natural products. The model was pre-trained on a processed ChEMBL dataset and then fine-tuned on a natural product dataset. Benefitting from transfer learning and the data balancing technique, the model achieved a highly promising area under the receiver operating characteristic curve (AUROC) score of 0.910, with limited task-related training samples. Since the embedding distribution difference is reduced, embedding space analysis demonstrates that the model’s outputs of natural products are reliable. Case studies have proved our model’s performance in drug datasets. The fine-tuned model can successfully output all the targets of 62 drugs. Compared with a previous study, our model achieved better results in terms of both AUROC validation and its success rate for obtaining active targets among the top ones. The target prediction model using transfer learning can be applied in the field of natural product-based drug discovery and has the potential to find more lead compounds or to assist researchers in drug repurposing.

## 1. Introduction

Natural products have long been an important source of drug discoveries. Among all the drugs approved since 1981, more than 60% have been related to natural products. These can include drugs that have natural product structures or leads derived from natural product scaffolds [1]. The difference between natural products and the molecules synthesized by chemists is obvious. For example, the possession of higher molecular weights and bigger scaffolds is more common in natural products. The scaffolds of natural structures are products of evolution [2]. As a result, there is often a greater probability of finding molecules that inhibit a series of expected targets in natural products [3,4]. Medicinal chemists have benefited from the identification of natural products [5], but the lack of bioactivity data on natural products remains an obstacle in drug discovery and drug design. In addition, a great deal of effort is required to clarify the interactions between compounds and protein targets via in vitro experiments.

In recent years, the use of in silico target prediction methods for small molecules has become popular [6]. Researchers have built powerful tools for both ligand-based and target-based target prediction [7,8,9]. With the evolving performance of graphics processing units (GPUs), deep learning methods have been adjusted for target prediction [10]. ChEMBL features 2M compounds and 17M activities [11], and therefore, it has become the most widely used dataset for data mining. In a previous study [12], various types of machine learning algorithms were evaluated on the same benchmark dataset. Methods based on feedforward neural networks outperformed those based on graph convolutional networks and other machine learning algorithms among a variety of molecular representations to achieve the highest AUROC score of 0.743. By adding more neural layers, a deep learning method can extract more task-related embedding features from the input [13] than traditional machine learning methods. On the other hand, the deep learning algorithms depend more on the amount and quality of the training data.

Though researchers have been discovering natural products for decades, and a large number of natural products have been identified [14,15,16], there are not enough natural product target data. This makes training a deep learning model challenging; transfer learning can be a solution for this issue [17]. Transfer learning deals with a pair of machine learning models that share similar tasks or distribution patterns [18]. Transfer learning can be viewed as a technique to minimize the mathematical distance between the source task and the target task. Researchers have already used the transfer learning technique to solve the inadequacy of data accomplished highly impressive outcomes [19,20,21,22,23]. Finetuning is a frequently used technique among property prediction tasks [21] and molecular generation tasks [23]. It is also one of the most adaptable methods and requires less hyperparameter optimization, which means that it requires less time to train.

In previous studies, the prediction of natural product bioactivities was commonly performed by models trained on datasets containing both human synthetic compounds and natural products [24,25,26]. Chen et al. [27] managed to use structural information from conventional simple molecules to predict targets for natural products and macrocyclic ligands. Even though these models can achieve fairly high accuracy in a test set, their predicted results for natural products remain unsubstantiated. Knowledge about natural products themselves has not been extracted or learned. The difference between the structures of natural products and those of synthetic compounds requires different ways to predict targets. A prediction model that specially targets natural products is in demand but has not been developed yet.

In this study, we adapt the transfer learning method to a deep learning algorithm, multilayer perceptron (MLP), in order to predict the targets of natural products. To apply our training methods to natural products, we first train a deep learning model on the ChEMBL dataset with natural products removed. With the knowledge from a much larger dataset, the model can learn the basic relationship between structures and targets. Then, the model is fine-tuned using a natural product dataset with a higher learning rate and some of the parameters frozen. After this step, the model acquires the ability to predict targets of different compounds and to adjust its parameters to fit the specific distribution of natural products. The AUROC score showed that the transfer learning step optimized the model’s performance on the natural products test set. The model can be used as a powerful tool to assist drug design based on the structure of natural products.

## 2. Results and Discussion

### 2.1. Hyperparameters Optimization of the Pre-Trained Model

**Figure 1:**
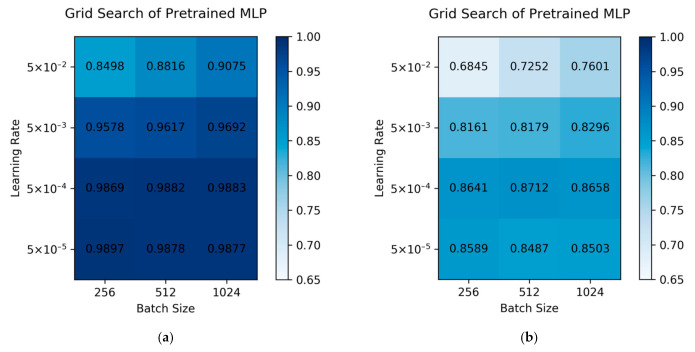
(**a**) Grid search for the pre-trained network over a fivefold cross-validation method. (**b**) Grid search for the pre-trained network over the intersection of the COCONUT dataset and the ChEMBL dataset. The values in the grids are the mean AUROC scores. The higher scores our models have achieved are colored with a deeper blue background. A score of 0.65 is defined as a baseline of the transparent color.

The hyperparameter optimization step of a deep learning model is crucial to its performance. The MLP algorithm may suffer from a set of bad hyperparameters, resulting in poor accuracy or overfitting. To test pre-trained MLP models, the fivefold cross-validation method is applied. The average performance is summarized in Figure 1a. Learning rates have a great influence on our models. We can conclude that among all the learning rates we tested, the smaller our learning rates were, the higher the AUROC the model could achieve. The reason for this phenomenon is that a large learning rate might cause a drastic fluctuation near the minimum loss function value, though it might avoid dropping into the saddle points. However, a small learning rate is more stable but takes more training time to converge. When the learning rates are around 5 × 10^−4^ to 5 × 10^−5^, there is no statistical difference in the model performances. The models that possess learning rates of 5 × 10^−4^ and 5 × 10^−5^ also achieve the highest AUROC score among all the sets of hyperparameters. Therefore, these low learning rate models are selected as candidates, because these models have learned the knowledge from a nearly full-size ChEMBL dataset well. The batch size’s influence was found to be slight when we viewed the validation sets.

Besides the prediction performance in the validation sets, we also ran these models on the natural product dataset, which is presented in Figure 1b. The models that possess a learning rate of 5 × 10^−4^ outperform the models that possess a learning rate of 5 × 10^−5^. Though the best model, which has a batch size of 512 and a learning rate of 5 × 10^−4^, achieved a fairly high AUROC of 0.87, we could not choose this set of hyperparameters for further finetuning. Only the results of the validation sets were allowed to be used for choosing hyperparameters. However, these AUROC scores did illustrate that our pre-trained MLP models had already achieved a decent performance in the natural product dataset.

### 2.2. Effectiveness of Transfer Learning

Once the model was pre-trained on the ChEMBL dataset from which the natural products were excluded, we applied the fine-tuning strategy on our pre-trained models. Learning rates ranging from 5 × 10^−2^ to 5 × 10^−3^ searches were applied in this step, and batch sizes of 32, 64, and 128 were tested. Only learning rates larger than 5 × 10^−4^ were applied, because the models needed to acquire more knowledge from the natural product dataset. The same learning rates as the pre-trained models would limit our models in exploring the novel distribution of chemical space.

**Figure 2:**
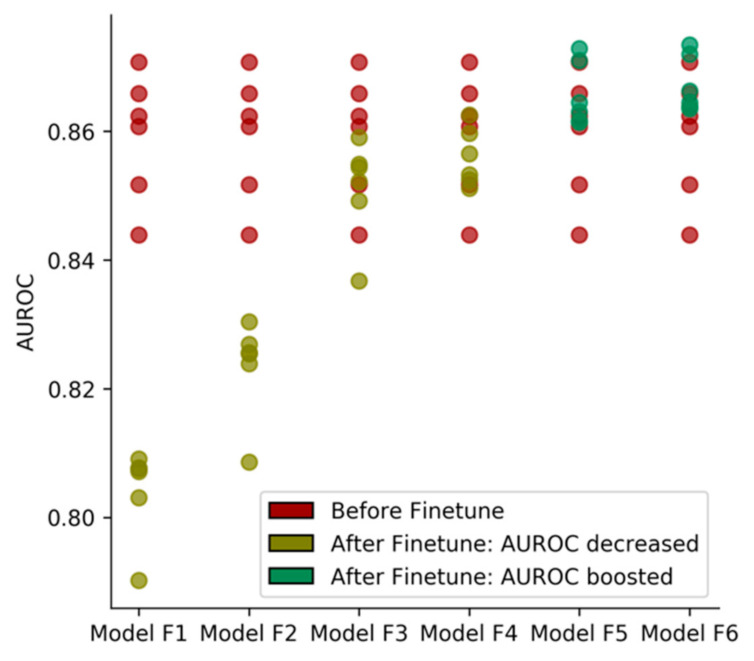
The effect of the use of different sets of transfer learning hyperparameters. Models F1–F6 correspond to the model finetuning with learning rate: 5 × 10^−^^2^, batch size: 32; learning rate: 5 × 10^−2^, batch size: 64; learning rate: 5 × 10^−2^, batch size: 128; learning rate: 5 × 10^−3^, batch size: 32; learning rate: 5 × 10^−3^, batch size: 64; learning rate: 5 × 10^−3^, batch size: 128.

One of these sets of hyperparameters (learning rate: 5 × 10^−3^, batch size: 128) attained the best AUROC in the test set based on our six candidate pre-trained models, hence it was chosen for further validations and case studies. Figure 2 shows that batch size has an influence on the model’s performance. A larger batch size usually leads to a higher AUROC score.

**Figure 3:**
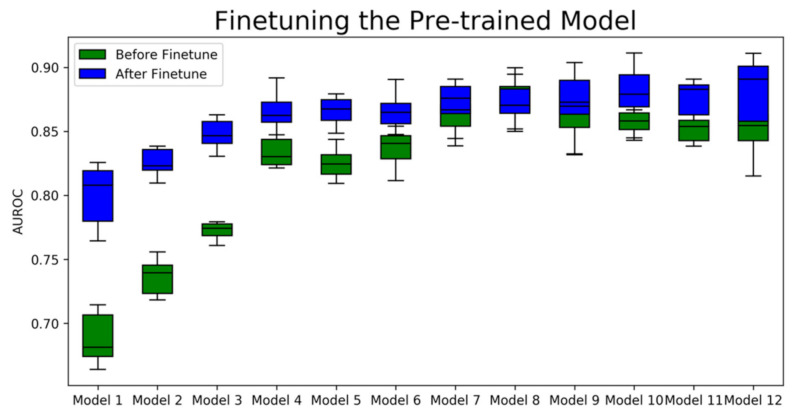
The average AUROC of the pre-trained models and fine-tuned models of the test set. Model 1 to model 12 are models that possess different sets of hyperparameters listed in Table 1. The green boxes are the distributions of AUROC scores computed from the random split test sets before finetuning the pre-trained models, and the blue boxes are the distributions of AUROC scores computed from the same random split test sets after 100 epochs of finetuning.

As shown in Figure 3, nearly every models’ AUROC score is boosted, compared to the pre-trained ones. The pre-trained models have poor performance benefits over a 0.1 AUROC boost effect and the candidate models that we selected obtained at least 0.01 promotion, except for model 8 and model 9. Model 8 achieved the highest AUROC among all the pre-trained models but showed a decrease in the finetuning step. The decrease infers that Model 8 overfits the dataset that does not include natural product bioactivities. Among all the fine-tuned models, model 12 achieved the highest average AUROC. As a result, we selected model 12, which possessed a learning rate of 5 × 10^−5^ and a batch size of 1024 during pre-training for further study.

We can also discern that the degree of promotion obtained has a close relationship with the split of the training and test datasets. This result was observed when we split the natural product dataset randomly 10 times at a ratio of 0.9:0.1. The box plot reflects the distribution of the models’ performances. For example, in model 12, which achieved the best mean AUROC score, the AUROC varied from 0.85 to 0.92. These outcomes result from the limited amount of training data and low diversity of the labeled natural product structures, compared to the full-sized ChEMBL dataset. Though the fine-tuning step has a promotive effect on the pre-trained models, it is wise to run the finetuning code several times and select the one with the best AUROC because of a more diverse training set. In our trial of finetuning model 12, nine models achieved a higher AUROC than the original model, and two of them achieved an AUROC higher than 0.9.

We treated the targets as “active targets” if the predicted probability was larger than 0.5. By setting a threshold value, we were able to calculate other validation standards to evaluate the effect of transfer learning. As shown in Table 2, the fine-tuning step showed an improvement in the AUROC and SE criteria. SE is important when we verify the model’s performance, and promotion to 0.6394 ± 0.005909 was an impressive outcome. More SE comparison can be seen in Section 2.4. From this result, we can see that the fine-tuned model had an increased ability to detect active interactions. In contrast, all the criteria related to the false-positive rate suffering a decrease after the fine-tuning. The high SP and ACC scores of pre-trained models decreased slightly after applying the transfer learning technique, while the PR score dropped severely. This means that our model is more aggressive—that is, the model tends to give more positive predictions. From these results, it appears that our fine-tuned model has a higher false-positive rate in the natural product dataset. This outcome results from an inborn error of the deep learning target prediction algorithm because not all zeros in the binary labeled vectors are from true inactive bioactivity measurements [28]. In fact, the false-positive predictions might also include interactions existing in reality that have not yet been discovered. Since it is more challenging to synthesize and test the bioactivities, this offset has a higher probability in the natural product dataset. For the above reasons, A low PR value was acceptable in this circumstance. The standards that account for experimentally proven interactions should be emphasized when considering the model performance. In addition, the researchers who work with drug repurposing can take advantage of this model feature to discover new targets for existing drugs. When we used a drug dataset that is extracted from natural products, we discovered a similar result. The sensitivity of the model was improved, but the PR score dropped. We also carry out a case study on the bad case addressing this high false-positive issue in Section 2.5.

**Figure 4:**
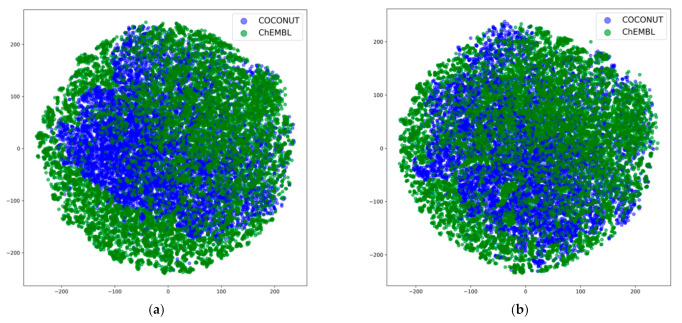
(**a**) Dimensionality reduction image of the pre-trained model embedding space and (**b**) dimensionality reduction image of the fine-tuned model’s embedding space. The two-dimensional space is generated by a step-wise algorithm. More details of the dimensionality reduction method can be found in Section 3.4.

The interpretability of deep learning remains a problem to be solved. The reason why transfer learning has the ability to improve deep learning models is a topic that we are interested in. In the fine-tuning step, the trainable parameters were from the batch normalization layers (Batch Norm) and the classifier layer. Adjusting the Batch Norm can achieve the transfer of data distribution, similar to the concept of adaptive batch normalization (AdaBN)) [29], which has been proven to function in computer vision benchmarks. On the other hand, the natural product data are still extracted from the ChEMBL dataset; therefore, whether our model can be applied on a larger natural product dataset such as the whole COCONUT dataset is uncertain. We looked into the embedding space and performed a dimensionality reduction method for these 1024-dimensional vectors, as plotted in Figure 4. When we extracted the embedding space at the time we applied our pre-trained model, it was obvious that a portion of the embedding space of the natural product dataset was not included in ChEMBL. The chemical space corresponding to this area was not seen by the pre-trained model. However, in the dimensionality reduction figure of the fine-tuned model, the space of the dataset that was labeled with bioactivities was expanded. The area that had target information covered more space from the 40 k natural products. The domain confusion loss between the embedding space, which usually comes from the knowledge difference of tasks, was reduced. The adaption of Batch Norm revealed the principle of transferring source knowledge to target tasks. This result lends credence to applying our fine-tuned model in larger natural product datasets.

### 2.3. Data Balance

**Figure 5:**
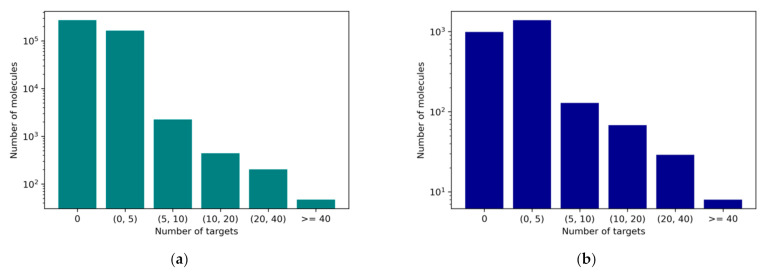
(**a**) Number of molecules with a certain number of targets in the ChEMBL dataset and (**b**) number of molecules with a certain number of targets in the intersection of the ChEMBL dataset and COCONUT dataset.

The imbalance of a dataset occurs when the majority category outnumbers the minority category to an unacceptable degree. In this case, the micromolecular structures that are active toward a protein target are far fewer than the inactive ones. Listed in Figure 5, more than half of the compounds have fewer than two active high-frequency targets recorded in both ChEMBL and COCONUT datasets. The distribution of the target number of these two datasets is similar, which gave us the confidence to apply the same model structure and data processing methods. If there was no technique applied to solve the imbalance situation, our model would tend to predict active targets as inactive just to minimize the training loss on the majority categories and would not be able to learn the relation between small molecules and single proteins.

**Figure 6:**
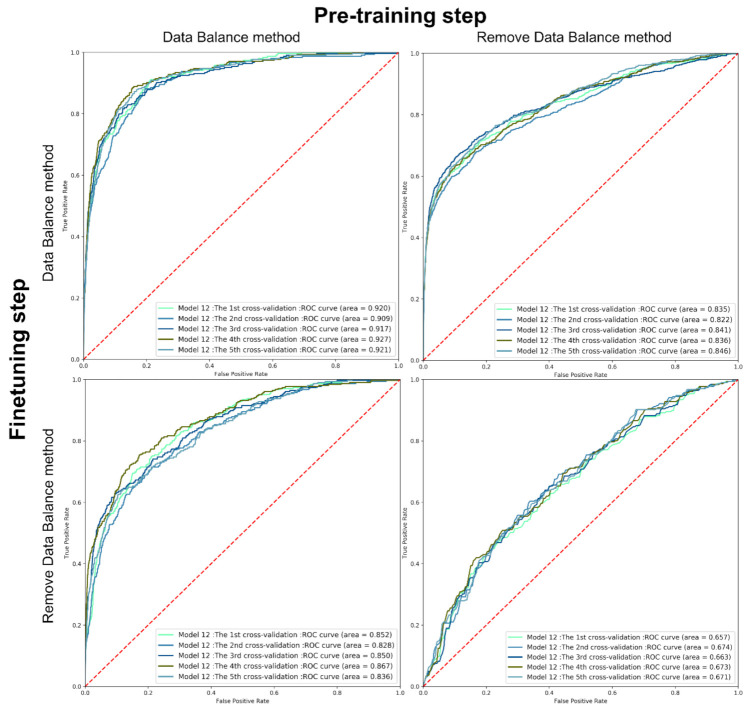
AUROC curve of models removing the data balancing method in the pre-training and fine-tuning step.

With the help of data balancing, the model can reach a higher accuracy in predicting active targets rather than being conservative. To prove this opinion, we removed the data balance methods in different steps in the model training to see whether weighing the loss function according to the activity data was helpful. The four different models refer to the training process of pre-training with/without data balancing and fine-tuning with/without data balancing. They were tested on the same random split natural product dataset. As shown in Figure 6, the AUROC scores dropped drastically, compared with the model going through data balancing in both steps. When we removed the weighing process in the fine-tuning step, the AUROC decreased from 0.92 to 0.85. Meanwhile, removing the data balancing method in the pre-training step caused a decrease of 0.09 in the AUROC. The receiver operating characteristic (ROC) curve shows that the models had poor early recognition abilities when we removed the data balance method in both steps. The AUROC decreased drastically to lower than 0.7.

In general, data balancing plays an irreplaceable role in pre-training and fine-tuning our model on ChEMBL. Moreover, this type of dataset is commonly seen in the medicinal chemistry field—for example, in virtual screening datasets. It is always necessary to implement a data balancing technique when dealing with datasets with this distribution.

### 2.4. Comparison with References

A natural product target prediction model called STarFish [26] was developed in a previous study. STarFish stacked several machine learning models, including K-nearest neighbors (KNN), random forests (RFs), and MLP. It was trained on ChEMBL, with natural products excluded. The researchers claimed that with the power of stacking, it was able to identify targets for natural products. The model managed to reach high accuracy on the natural product benchmark. To test our fine-tuned model further, a comparison was executed.

**Figure 7:**
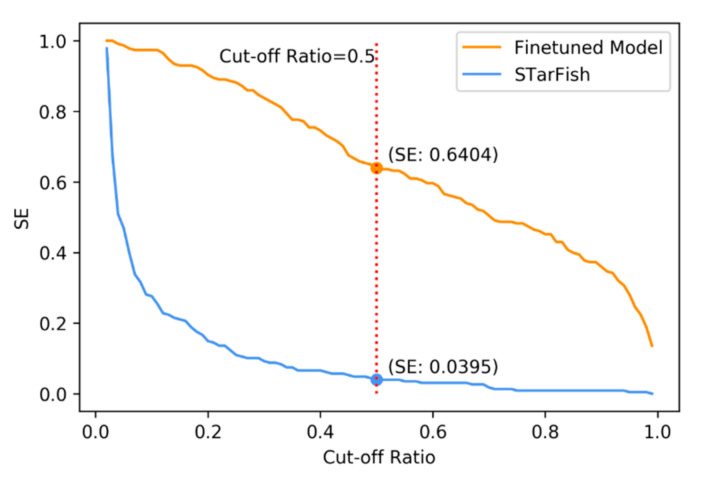
The effect of different cutoff ratios on the SE. The two curves correspond to the STarFish model and the fine-tuned model. The validation is carried on the same test set.

The number of targets both models were able to output possibilities for was 224, which means that five of our high-frequency targets were not found in STarFish’s training set. This might have occurred because of the ChEMBL dataset’s version updates. When comparing the performance in these shared targets, our model outperformed STarFish in terms of both AUROC and the probability of possessing at least one active target among the top 15/20 targets, as listed in Table 3. We can also infer that the AUROC is not sensitive in comparison with other interpretable standards. Though there is only a slight difference between the AUROC scores, the possibilities of obtaining active targets among the top ones differ vastly. Sensitivity is another intuitive validation method for target prediction. SE refers to the probability of detect positive interactions. A high SE score demands that the model attaches high probabilities to positive interactions instead of simply getting a higher rank. SE is more informative than the probability of having active interactions in the top 15/20. We conducted a comparison of SE between STarFish and our model. The SE value depends on the choice of cut-off ratio, so we drew curves showing the SE values in a large range of cutoff ratios (see Figure 7). A value of 0.5 is the most common choice for the cutoff ratio. When 0.5 is chosen for testing, our model achieves a score of 0.6404 and STarFish obtains a score of 0.0395. Though predicting targets for natural products is a complex task, our fine-tuned model reached an impressive outcome. The model based on datasets containing only human synthesized compounds tended to give low probability scores for positive targets.

### 2.5. Case Study

In order to test the transfer learning-based model in the real drug design field, we carried out a series of case studies.

**Figure 8:**
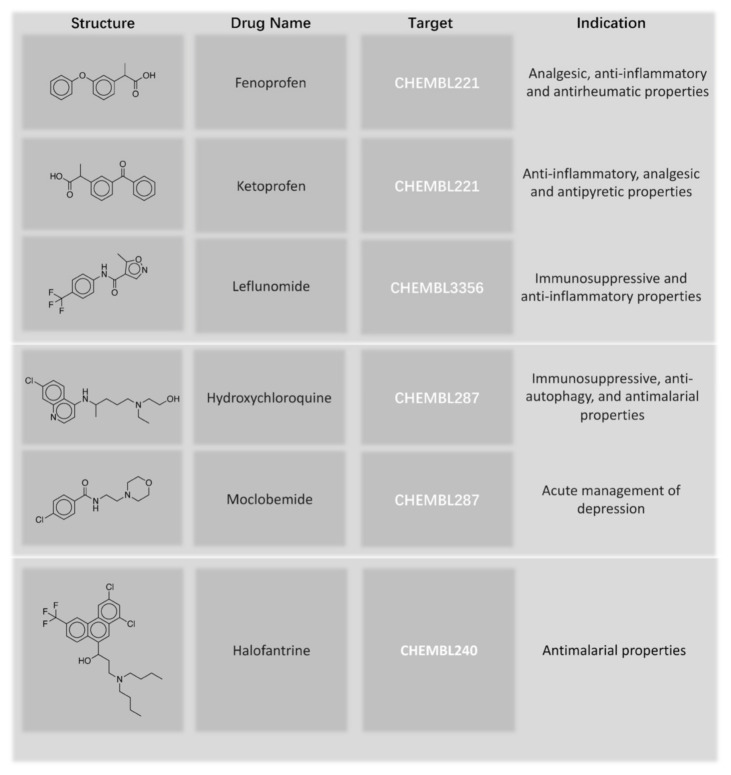
Structures of the approved drugs that the fine-tuned model predicted all the right targets for. The drug names and indication information are from DrugBank.

First, we removed the drugs from the natural product training set and retrained the model. Then, we tested our model on the approved drug dataset. Among 139 approved drugs whose structures were directly from natural products, our fine-tuned model predicted every active target of 62 drugs, including both single-target drugs and multi-target drugs when we set the cutoff ratio to 0.5. Meanwhile, the pre-trained model predicted all the active targets of 56 drugs. Among all the drugs that our fine-tuned model was able to predict every active target for, the drug with the most targets was Fluphenazine. It possesses 15 targets, and our model successfully output all 15 experimentally measured targets and gave 19 more recommended targets (see Table 4). The recommended targets marked with asterisks are listed as active in the ChEMBL web interface. These false-positive predictions marked with asterisks include (1)targets that had IC50/Ki/Kd values higher than 1000 nM but lower than 3000 nM (2) targets that have been tested in multiple assays, which have both values higher than 1000 nM and values lower than 1000 nM. This result shows that the false-positive predictions are valuable for researchers who work on drug repurposing. When the compounds possessed fewer active targets, the model performed very well. As shown in Figure 8, various types of drug targets can be predicted. The structures listed were selected from the approved drug dataset. Three drugs that had anti-inflammatory properties, two drugs that inhibited the sigma opioid receptor, and an antimalarial drug were found to have structures derived from natural products. All of these drugs’ targets can be successfully predicted by our model. The full predicted results for the approved drugs are listed in Supplementary Table S1. The probability of obtaining an active target in the top 15 predicted targets is 74.1%. When we expand to the top 20 highest probability targets, the ratio of our model’s success in having at least one active target among the top targets is 78.4%.

**Figure 9:**
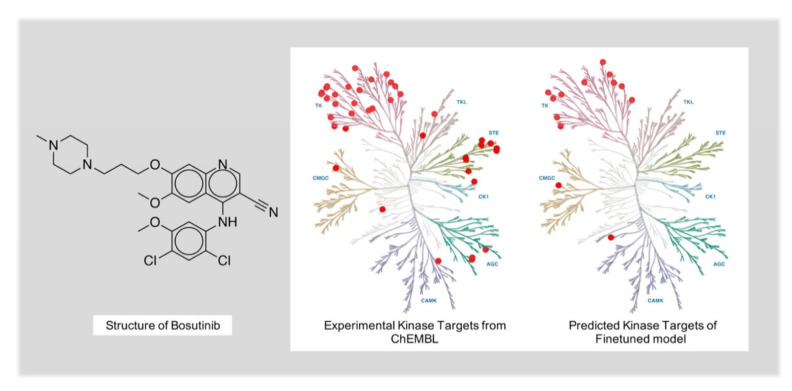
The experimental kinase targets and predicted kinase targets of Bosutinib.

Though the model performed well when making predictions for drugs with a few targets, the model was not able to perform satisfactorily for drugs with a low target selectivity. Therefore, we went through the bad cases. For example, Bosutinib, which is the approved drug that has the maximum number of high-frequency active targets in our test dataset, had 40 active targets. The fine-tuned model only successfully predicted six of them. The active targets of Bosutinib are all from the kinase family, and 13 predicted targets are from this protein family. With the help of Kinmap, an online tool to visualize the kinase family, we can see that the real targets are distributed in all kinase families, while the predicted targets only cover three kinase families (see Figure 9). This may due to the lack of multi-target samples in the training data (see Figure 5), leading to the drop in the success rate of the model in molecules with tens of targets. However, it is worth noting that almost all the predicted kinase targets were distributed in the tyrosine kinase(TK) family. This selectivity toward the TK family is consistent with the experimental kinase selectivity. The six targets correctly predicted by the model also belonged to the TK family. This indicates that our fine-tuned model had already learned the relation between drugs and targets, even in the bad cases.

## 3. Methods

### 3.1. Preparation of the Dataset

The ChEMBL27 dataset [11] and COCONUT dataset [30] were used to generate the dataset to train and validate the natural product target prediction model. ChEMBL27 is the source of bioactivity data. The assays that contained the activity type IC50, EC50, Ki, or Kd and target type “SINGLE PROTEIN” were extracted. When there was more than one assay recording the same compound’s standard bioactivity values toward a target, average values were computed. If the IC50/EC50/Ki/Kd value was less than 1000 nM, this compound–target interaction was converted as an active target site and vice versa. COCONUT is the source of natural product structures. In both COCONUT and ChEMBL27, structures containing atoms other than {H, C, N, O, P, S, F, Cl, Br} were removed. A sanitizer from the python third-party package RDkit [31] was used to remove charges and chirality and keep the largest fragment. If the structures were the same after these chemical sanitizing procedures but did not have the same active targets, both of them were removed.

When the above data cleaning was completed, there were 700 k molecules in the ChEMBL27 dataset. All the unrecorded compound–target interactions were automatically labeled as inactive. We removed all the compounds with no active targets and all the targets with no active compounds. Then, we obtained a data chart containing the active/inactive information of 450 k molecules toward 4193 targets.

The structures from ChEMBL27 and COCONUT were intersected to obtain the bioactivities of 2600 natural products. The intersection was removed from the cleaned ChEMBL27 dataset. In this way, the bioactivities chart was divided into a dataset that contained no natural products and a dataset consisting of natural products. The former one was used to pre-train the model, and the latter one was used to fine-tune the model. When we observed the targets of these 2600 natural product structures, we found that most of the targets only possessed few active structures. A prediction model will not learn a strong abstracting ability if the quantity of labeled positive data is excessively low, and we wanted our model to focus on the targets that had a high recognition. As a result, we removed the low-frequency targets that possessed less than 10 active compounds in the natural product dataset. A total of 229 high-frequency natural product targets were left to build the model. Detailed information about these targets can be viewed in Table S2. Structures that were only active to the low-frequency targets were retained.

### 3.2. Model Structure

**Figure 10:**
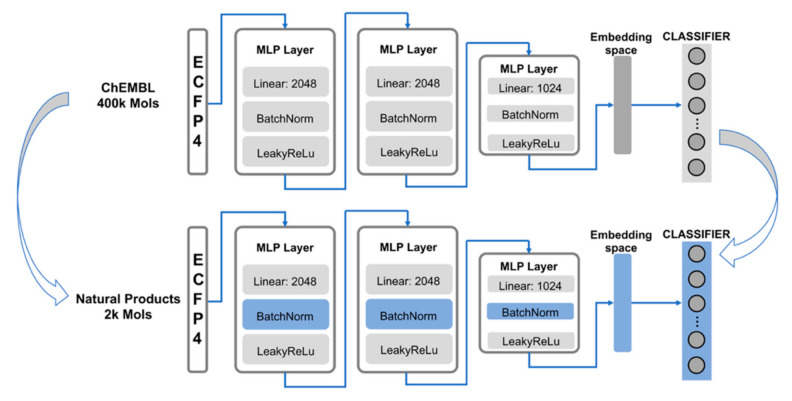
Model structure of the target prediction model.

MLP is a structure that has been widely used to complete multitask deep learning work. The hidden layers are able to capture the shared embedding space and thereby learn the relations between tasks. In a previous study [12], the structure of MLP outperformed other ECFP fingerprint-based machine learning algorithms, such as support vector machine (SVM), KNN, RF, Naïve–Bayes statistics (NB), and similarity ensembles approach (SEA), as well as graph-based algorithms such as graph convolutional network (GCN) when applied in multi-target prediction tasks. We built a similar model structure to predict the high-frequency targets of natural products, as shown in Figure 10.

All the canonical smiles of the compounds were converted to extended connectivity fingerprints with a radius of 2 (ECFP4) and a binary bit size of 2048. These fingerprints were fed into the model as an input, representing the two-dimensional structures of the compounds. Batches of 2048 bit vectors were processed by three capsules of MLP layers. Each capsule contains different sizes of linear layers, a Batch Norm layer, and a leaky rectified linear activation unit (RELU) layer. The Batch Norm layer and leaky RELU layer acted as nonlinear activation functions and avoided overfitting the training dataset. The embedding space is the input of the classifier layer, which resizes the vectors with a linear layer and normalizes the value range from 0 to 1 with the sigmoid function. The output of the classifier layer is a vector of size 229, representing the probability of being active among the high-frequency targets.

### 3.3. Pre-Training and Transfer Learning

As the epoch of training increases, the loss between outputs and labels decreases. Nonetheless, the model’s accuracy on the test set will be reduced because of the overfitting of the training set. Therefore, a random split fivefold cross validation was applied in the pre-training step. The ChEMBL dataset with the natural products removed was divided into five folds. One of the folds was left out each time as a validation set. Once the AUROC score in the validation set had decreased five times, the training would be stopped. The AUROC score was calculated every five epochs. All the parameters in the neural networks were accessed to be updated during the pre-training step.

In the transfer learning step, fine-tuning was the technique we implemented. With a smaller amount of data being used for training, a portion of the parameters was always frozen to keep the abstract knowledge learned from the source dataset stable. The linear parameters in the hidden layers were frozen in this case. The Batch Norm parameters and the linear parameters in the classifier layer were accessed for the optimizer to update. The Batch Norm parameters worked as a mathematical treatment to minimize the difference between the pre-training dataset and fine-tuning dataset, while the classifier parameters were fine-tuned to learn more about the specific embedding space of the natural products. A higher learning rate was also applied. To make the most of our natural product dataset, a random train–test split with a ratio of 0.9:0.1 was used. Due to the size of the dataset, the iteration time was fixed to 100. The finetuning step converged in a short period of time, hence observing the accuracy decrease in the validation set to determine the time to stop training was unsuitable.

In both pre-training and fine-tuning, the optimizer Adam and loss function binary entropy loss were applied to train the parameters of the neural network. The momentum hyperparameter β1 was fixed to 0.9 and β2 to 0.999. Additionally, a weight decay of 0.002 was used in every hidden layer parameters backpropagation process to avoid overfitting. The learning rate and the size of the minibatch are listed in Table 1 for pre-training.

### 3.4. Dimensionality Reduction

Dimensionality reduction is a widely used technique to visualize the knowledge neural networks learned from the dataset. At first, a principle component analysis (PCA) was applied to extract the features from the embedding space. We kept selecting the eigenvectors until the sum of the corresponding eigenvalues added up to 0.9 of the whole sum of the 1024 eigenvalues. In this way, a 1024 high-dimensional embedding space was compressed into a 40-dimensional PCA space. To compress more in order to visualize it in a 2D image, T-distributed stochastic neighbor embedding(t-SNE) was applied to reduce the dimension of the embedding space further. Iterating the t-SNE for 1000 epochs, this method yields tuples containing two floating points for each molecular structure. The new distribution is visualized in Figure 4. The two axes represent the compressed dimensions of our embedding space computed by this algorithm. A total of 2% of the molecules of each dataset were randomly sampled to plot the dimensionality reduction image.

### 3.5. Data Balance

As shown in Figure 5, imbalanced data occurs in our datasets. Therefore, a cost-sensitive method [32] was applied to address this issue. Although oversampling methods have achieved a great performance in single task classification works [33], whether they can be applied in multitask imbalance situations is unclear. However, a multitask deep neural network (MTDNN) using a cost-sensitive method has been proven to be effective [34]. The cost matrix is crucial for the cost-sensitive method. Since we included structures that had no high-frequency targets but possessed low-frequency targets, we redesigned the matrix generating algorithm in the previous study. Our cost matrix was generated in the following pattern:

(1) $$if\;\overset{229}{\underset{j=1}{\sum }}label_{j}>0,\;\;cost_{j}=\left\{\begin{array}{c} N_{i}/N_{a},\;\;label_{j}=1\\ 1,\;\;label_{j}=0 \end{array}\right.\;\;\;\;if\;\overset{229}{\underset{j=1}{\sum }}label_{j}=0,\;\;\;\;\;\;\;\;\;\;cost_{j}=1.\;\;\;\;\;\;\;\;\;\;\;\;\;\;\;\;\;\;\;\;\;\;\;\;\;\;\;\;\;\;\;\;\;\;\;\;\;\;\;\;$$

where *label* is a 229-bit binary vector representing the bioactivity of the compound. *label_j_* stands for the binary value of the compound‘s activity toward the No.j target. *N_i_* and *N_a_* stand for the number of inactive targets and active targets of the compound. *cost* is the vector corresponding to the compound in the cost matrix. For short, the vectors of the compounds that have no high-frequency targets and the units that represent inactive targets are regulated as one. As for the units that represent active interactions, the higher the selectivity of the compound–target interaction is, the higher the cost generated will be.

### 3.6. Validation Methods

The target prediction model usually requires various types of validation methods to be carried out to verify its performance. The AUROC of the multitask model was defined as the average area under the ROC curves of all tasks. In this case, all the predicted probability vectors of the test dataset were merged, as well as the real activity vectors. These two long vectors were fed into the function of the ROC curve from sklearn [35] to compute the AUROC score. AUROC is a validation method that does not rely on the choice of cut-off ratio. This method also observes the positive accuracy rate and the negative accuracy rate at the same time. As a result, it is a fair measurement for classification models.

*SE*, *SP*, *PR*, *ACC*, and *MCC* are five other methods we used to validate our model’s performance. These quantities are directly derived from the confusion matrix, which represents different abilities of the models. The computational formulas are listed below.

(2) $$SE=\frac{TP}{TP+FN},$$

(3) $$SP=\frac{TN}{TN+FP},$$

(4) $$PR=\frac{TP}{TP+FP},$$

(5) $$ACC=\frac{TP+TN}{TP+TN+FP+FN},$$

(6) $$MCC=\frac{\left(TP\times TN\right)-\left(FP\times FN\right)}{\sqrt{\left(TP+FP\right)\times \left(TP+FN\right)\times \left(TN+FP\right)\times \left(TN+FN\right)}}.$$

In the equations, *TP* stands for the true-positive rate, *TN* stands for the true-negative rate, *FP* stands for the false-positive rate, and *FN* stands for the false-negative rate.

The natural product dataset in which the drugs’ structures have been removed were randomly split with the ratio of 0.9:0.1. Our pre-trained model was fine-tuned on the dataset containing 90% structures and the comparison between our model and STarFish was carried on the test set containing 10% structures. The stacked model of STarFish was directly downloaded from the reference’s Github pages [26]. None of the source codes were changed, except the output targets, which were specified to the targets both models contained. As mentioned above, the AUROC and SE were implemented in this situation to evaluate the performance.

### 3.7. Case Study

The structures from DrugBank [36] were downloaded to be analyzed with our natural products dataset, which was labeled with bioactivities. The structures of approved drugs and drugs including the compounds going through clinical trials were extracted from natural products by intersecting the DrugBank dataset with our natural product dataset. The intersection of the DrugBank dataset and our natural products dataset included 952 drugs whose bioactivities could be extracted from ChEMBL, and the structures were directly from natural products. Among them, 139 approved drugs possessed high-frequency natural product targets. The model used for the case studies was fine-tuned on a dataset in which all drug structures were removed. The cutoff rate to present the predicted targets was set to 0.5.

Kinmap [37] is the tool we used to analyze the bad case. This online tool features the convenience to visualize kinase proteins and the relation between protein families. Both the experimentally proven targets and the predicted targets of Bosutinib from the kinase family were interpreted into standardized kinase names. Then, the names were inputted into the interactive web page, and the kinase tree diagrams in Figure 9 were generated.

## 4. Conclusions

In this study, a novel method of target prediction for natural products was proposed. A multitask neural network based on transfer learning was trained with the ChEMBL dataset. The model was evaluated using various types of validation methods. Fine-tuning could boost the performance of the pre-trained model and also minimize the distribution difference between the training data and the natural product dataset. With the help of data balancing, the fine-tuned model achieved state-of-the-art AUROC scores and successfully predicted high-frequency targets of a certain number of approved drugs whose structures are from natural products. Natural products have been proven to provide potential druggability. Since a large proportion of natural products have not been tested, this method can be applied to discover novel lead compounds.

## Figures and Tables

**Table 1:** Grid search space of pre-trained models.

	Grid Search Space
Model 1	Learning Rate: 5 × 10^−2^ Batch size: 256
Model 2	Learning Rate: 5 × 10^−2^ Batch size: 512
Model 3	Learning Rate: 5 × 10^−2^ Batch size: 1024
Model 4	Learning Rate: 5 × 10^−3^ Batch size: 256
Model 5	Learning Rate: 5 × 10^−3^ Batch size: 512
Model 6	Learning Rate: 5 × 10^−3^ Batch size: 1024
Model 7	Learning Rate: 5 × 10^−4^ Batch size: 256
Model 8	Learning Rate: 5 × 10^−4^ Batch size: 512
Model 9	Learning Rate: 5 × 10^−4^ Batch size: 1024
Model 10	Learning Rate: 5 × 10^−5^ Batch size: 256
Model 11	Learning Rate: 5 × 10^−5^ Batch size: 512
Model 12	Learning Rate: 5 × 10^−5^ Batch size: 1024

**Table 2:** Validation scores of the pre-trained model and fine-tuned model. The values that were promoted are bolded in the table.

	AUROC	Sensitivity (SE)	Specificity (SP)	Precision (PR)	Accuracy (ACC)	Matthews Correlation Coefficient (MCC)
Model: Pre-trained model Test: Natural Products (drug removed)	0.8461	0.3842	0.9932	0.2884	0.9889	0.3274
Model: Pre-trained model Test: Natural Products (drug set)	0.8548	0.4167	0.9935	0.4158	0.9872	0.4098
Model: Fine-tuned model Test: Natural Products (random split 10% set)	**0.8849 ± 0.00097**	**0.6394 ± 0.005909**	0.9401 ± 0.000012	0.06617 ± 0.000113	0.9380 ± 0.000008	0.1908 ± 0.000319
Model: Fine-tuned model Test: Natural Products (drug set)	0.7646	**0.5321**	0.9159	0.0654	0.9117	0.1636

**Table 3:** Comparison between the fine-tuned model and STarFish.

	Probability of Top15	Probability of Top20	AUROC
Fine-tuned Model	0.817	0.860	0.910
STarFish	0.621	0.653	0.899

**Table 4:** Structure and targets of fluphenazine. The potential targets that appear in interactions weaker than our data cleaning standards are marked with asterisks (*) and bolded.

Structure of Fluphenazine	Predicted Experimental High-Frequency Targets	Recommended High-Frequency Targets
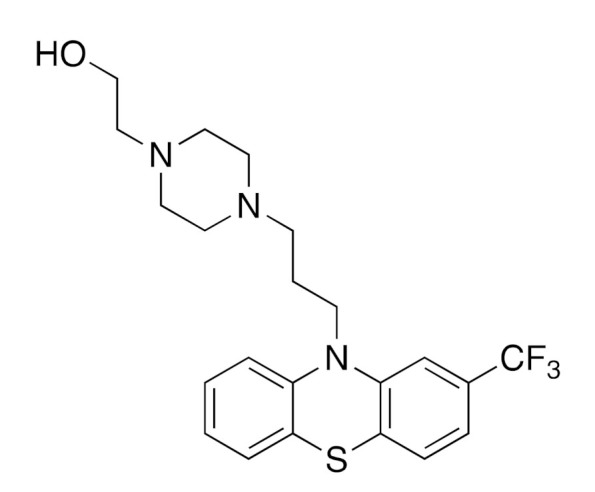	ChEMBL 217, ChEMBL 234, ChEMBL 224, ChEMBL 3371, ChEMBL 225, ChEMBL 287, ChEMBL 1833, ChEMBL 223, ChEMBL 231, ChEMBL 2056, ChEMBL 1867, ChEMBL 319, ChEMBL 1916, ChEMBL 1942, ChEMBL 315	ChEMBL 214, ChEMBL 273 *, ChEMBL 228 *, ChEMBL 339, ChEMBL 313, ChEMBL 222 *, ChEMBL 219, ChEMBL3155, ChEMBL 229, ChEMBL 322, ChEMBL 245 *, ChEMBL 216 *, ChEMBL 211 *, ChEMBL 3602, ChEMBL 265, ChEMBL 3943, ChEMBL 2035 *, ChEMBL 6007, ChEMBL 4081

## Data Availability

The data and code of this study are available from the corresponding author upon request.

## Supplementary Materials

The following are available online at https://www.mdpi.com/article/10.3390/ijms22094632/s1, Table S1: Predicted and experimental targets of approved drugs, Table S2: Information of high-frequency natural product targets.
